# Organizing and running bioinformatics hackathons within Africa: The H3ABioNet cloud computing experience

**DOI:** 10.12688/aasopenres.12847.2

**Published:** 2019-08-07

**Authors:** Azza E. Ahmed, Phelelani T. Mpangase, Sumir Panji, Shakuntala Baichoo, Yassine Souilmi, Faisal M. Fadlelmola, Mustafa Alghali, Shaun Aron, Hocine Bendou, Eugene De Beste, Mamana Mbiyavanga, Oussema Souiai, Long Yi, Jennie Zermeno, Don Armstrong, Brian D. O'Connor, Liudmila Sergeevna Mainzer, Michael R. Crusoe, Ayton Meintjes, Peter Van Heusden, Gerrit Botha, Fourie Joubert, C. Victor Jongeneel, Scott Hazelhurst, Nicola Mulder

**Affiliations:** 1 Centre for Bioinformatics and Systems Biology, Faculty of Science, University of Khartoum, Khartoum, Sudan; 2 Department of Electrical and Electronic Engineering, Faculty of Engineering, University of Khartoum, Khartoum, Sudan; 3 Sydney Brenner Institute for Molecular Bioscience, University of the Witwatersrand, Johannesburg, South Africa; 4 Computational Biology Division, Integrative Medical Biosciences, University of Cape Town, Cape Town, South Africa; 5 Department of Digital Technologies, University of Mauritius, Reduit, Mauritius; 6 Australian Centre for Ancient DNA, University of Adelaide, Adelaide, Australia; 7 South African National Bioinformatics Institute, University of the Western Cape, Cape Town, South Africa; 8 Institut Pasteur De Tunis and Institut Superieur des Technologies Médicales de Tunis, University Tunis Al Manar, Tunis, Tunisia; 9 Institute for Genomic Biology, University of Illinois at Urbana-Champaign, Urbana, IL, USA; 10 Genomics Institute, University of California, Santa Cruz, Santa Cruz, CA, USA; 11 National Center for Supercomputing Applications, University of Illinois at Urbana-Champaign, Urbana, IL, USA; 12 Common Workflow Language Project, Vilnius, Lithuania; 13 Centre for Bioinformatics and Computational Biology, Department of Biochemistry, Genetics and Microbiology, University of Pretoria, Pretoria, South Africa; 14 School of Electrical & Information Engineering, University of the Witwatersrand, Johannesburg, South Africa

**Keywords:** Bioinformatics, hackathon, workflow, reproducible, pipeline, capacity building

## Abstract

The need for portable and reproducible genomics analysis pipelines is growing globally as well as in Africa, especially with the growth of collaborative projects like the Human Health and Heredity in Africa Consortium (H3Africa). The Pan-African H3Africa Bioinformatics Network (H3ABioNet) recognized the need for portable, reproducible pipelines adapted to heterogeneous computing environments, and for the nurturing of technical expertise in workflow languages and containerization technologies. Building on the network’s Standard Operating Procedures (SOPs) for common genomic analyses, H3ABioNet arranged its first Cloud Computing and Reproducible Workflows Hackathon in 2016, with the purpose of translating those SOPs into analysis pipelines able to run on heterogeneous computing environments and meeting the needs of H3Africa research projects. This paper describes the preparations for this hackathon and reflects upon the lessons learned about its impact on building the technical and scientific expertise of African researchers. The workflows developed were made publicly available in GitHub repositories and deposited as container images on Quay.io.

## Introduction

As an inherently interdisciplinary science, bioinformatics depends upon complementary expertise from biomedical scientists, statisticians and computer scientists ^1^. This opportunity for collaborative projects also creates a need for avenues to exchange knowledge ^1^. Hackathons, along with codefests and sprints, are emerging as an efficient means for driving successful projects ^2^. They can be in the form of science hackathons that aim to derive research plans and scientific write up ^3^, community-driven software development ^4^, and data hackathons or datathons ^5^. In addition to the scientific and technical outcomes, these intensive and focused activities offer necessary skills development and networking opportunities to young and early career scientists.

**Figure 1.**
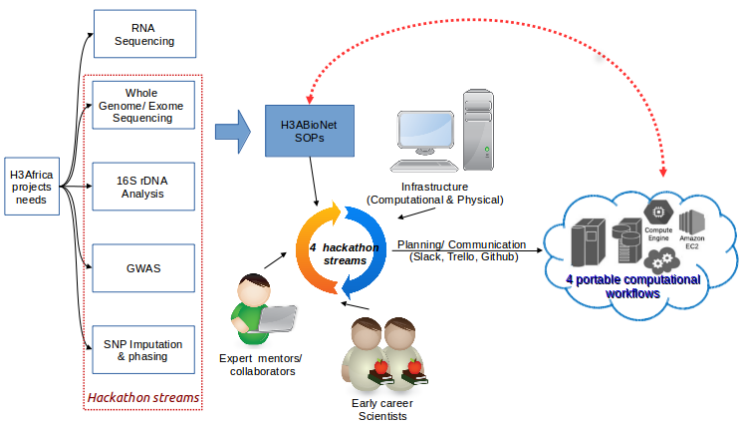
Planning and execution of the H3ABioNet Cloud computing hackathon. H3ABioNet developed SOPs for 5 analysis niches needed within H3Africa projects. 4 out of these were implemented as portable workflows as a result of the H3ABioNet 2016 Cloud Computing hackathon that brought together early career scientists, expert mentors and collaborators by utilizing many planning and communication platforms. (SOPs: Standard Operating Procedures).

On the African continent, there is generally limited access to such events. However, with the growing capacity for Africans to generate genomic data, the need to analyze these data locally by African scientists, is also growing. H3ABioNet ^6^, the Bioinformatics Network within the H3Africa initiative ^7^, has invested in capacity building via different approaches ^8^. The H3ABioNet Cloud Computing hackathon was a natural extension of the network’s efforts in developing Standard Operating Procedures (SOPs) via its Network Accreditation Task Force (NATF) ^9^; aimed at building and assessing capacity in genomic analysis. This also follows other efforts by the H3ABioNet Infrastructure Working Group (ISWG) towards setting up infrastructure at various H3ABioNet Nodes at the hardware, software, networking, and staff level. The H3ABioNet Cloud Computing hackathon, therefore, provided an excellent opportunity to assess the computational skills capacity development of the network through training, learning and adoption of novel technologies ( Figure 1). These technologies included workflow languages for reproducible science, containerization of software, and creation of computational products that can be used in heterogeneous computing environments encountered by African and international scientists in the form of standalone servers, cloud allocations and High-Performance Computing (HPC) resources.

In this paper, we discuss the organization of the H3ABioNet Cloud Computing hackathon, the interactions between the participants, and the lessons learnt. Baichoo *et al*. ^10^ describe the technical aspects of the pipelines, whereas the code and pipelines themselves have been made publicly available via H3ABioNet’s GitHub page in the following repositories: ( h3agatk, h3abionet16S, h3agwas and chipimputation) as well as container images hosted on Quay.io.

## Context, rationale and impact

For a healthy and strong scientific community, knowledge sharing activities, such as hackathons, are paramount. While instrumental to collaboration and efficient in developing solutions to shared problems, such activities are limited within Africa.

The H3ABioNet consortium aims to build a coherent and strong bioinformatics community within Africa that can technically support H3Africa projects for within-Africa analysis of African data. A network of > 27 nodes, H3ABioNet unites researchers from 15 African countries, in addition to a node in the US. Establishing a baseline where each node had sufficient computational infrastructure to carry genomics analyses was (and still) one of the key deliverables of the consortium. Consortium projects like Netmap helped to achieve this goal by evaluating network connectivity between the participating nodes and also led to upgrading infrastructure where warranted ^11^.

Consequently, the primary value of the H3ABioNet cloud computing hackathon was to expose African scientists to the practical aspects of community development of computer code and to try to create a community around the maintenance of a set of workflows that implement methods that are useful to the H3Africa research community and beyond.

More pragmatically, the workflows developed in the hackathon serve as practical implementations of the Standard Operating Procedures for the H3ABioNet Accreditation Exercises, which are used to evaluate the capacity of African research groups in analyzing complex genomic datasets- like those being produced by various H3Africa research projects ^9^. Success in taking one of the exercises is considered a landmark for African groups who are preparing to step into the existing gap between data production and data analysis, where the analysis is typically undertaken by First World groups.

Today, those implemented pipelines have been used for data analysis within the context of H3Africa projects, and/or incorporated into H3ABioNet training materials. Table 1 below highlights the significance of each developed pipeline, along with some technical notes about its implementation and availability. An extensive technical evaluation and trajectory of development is found in ^10^.

**Table 1.** Significance and impact of the developed pipelines as part of the H3ABioNet 2016 Cloud Computing hackathon, along with implementation notes.

Analysis pipeline	Implementation	Significance & Impact	Testing environment *	GitHub link **
Whole Genome/ Exome NGS Data Analysis	CWL	Such data is extensively generated within H3Africa projects (for example, the data informing the design of the African Genotyping chip ^15^ enriched by variants from 350 deeply sequenced African genomes)	• EGI FedCloud resource (+) • AWS ec2 (+/-) • Microsoft Azure VM (+/-)	https://github.com/h3abionet/h3agatk
16S rDNA Diversity Analysis	CWL and Nextflow	For performing 16S rDNA diversity analysis of microbial species in metagenomic samples (was derived from work done to analyze bacterial populations present in leg ulcers of sickle cell patients in Nigeria.)	• AWS EC2 & Azure VMs (+/-) • SGE cluster (+) • PBS cluster (-)	https://github.com/h3abionet/h3abionet16S
Genome-wide association studies (GWAS)	Nextflow	The H3Africa Consortium will genotype over 30,000 individuals using a custom designed African genotyping array. H3Africa projects, like AWI-Gen ^16^ have already extensively used this pipeline to analyze more than 11.5k samples at the time of writing. Additionally, this pipeline is now part of H3ABioNet training resources on GWAS, with online content readily available via ( https://www.youtube.com/playlist?list=PLcQ0XMykNhCQJPz0a mnbz9BPM4Bu0Nkgf); and also for in-person, “Bring your Own Data” workshops	• PBS cluster • the Bright Cluster Manager (-) • AWS EC2 (Docker Swarm and cloud-init)	http://github.com/h3abionet/h3agwas
SNP imputation	Nexflow	Of value in population structure and admixture studies. Eventually, this pipeline (along with computational resources from well-resourced H3ABioNet nodes) are intended to be provided as a service to African researchers. Currently, this pipeline too is part of H3ABioNet training resources on GWAS, with online content readily available via ( https://www.youtube.com/playlist?list=PLcQ0XMykNhCQJPz0a mnbz9BPM4Bu0Nkgf); and also for in-person, “Bring your Own Data” workshops	• SGE cluster (-) • OpenStack cloud (+)	https://github.com/h3abionet/chipimputation/

* + and - indicates testing with and without docker, respectively, in the given environment

** Corresponding docker containers are available at: https://quay.io/organization/h3abionet_org and https://dockstore.org/workflows/h3abionet/h3agatk

## H3ABioNet Cloud Computing Hackathon Activities

Prior to the H3ABioNet Cloud Computing hackathon, H3ABioNet, via its Infrastructure Working Group (ISWG), formed a Cloud Computing task force to investigate cloud computing technologies, familiarize H3ABioNet members with current cloud implementations and gauge their suitability for H3Africa data analyses. The H3ABioNet Cloud Computing hackathon was one of the first deliverables of this task force, with the specific objective to test and implement four analysis workflows that can be ported on multiple computing platforms. Figure 1 shows this hackathon within the broader H3Africa context and provides a broad overview of the planning and execution of this activity, with details in the following subsections.

### Pre-hackathon preparations

The computational pipelines put forward for development during the H3ABioNet Cloud Computing hackathon were identified based on the data being generated by different H3Africa projects and the SOPs used for the H3ABioNet Node Accreditation exercises. Reproducibility and portability were also identified as key features for the workflows, due to the heterogeneous computational platforms available in Africa. H3ABioNet Nodes that used or helped develop current H3ABioNet workflows and SOPs were part of the planning team, as well as other nodes that had technically strong scientists who were willing to extend their skills.

In the course of planning for the H3ABioNet Cloud Computing hackathon, two technical areas were identified where additional expertise was required. These were containerization technology such as Docker, and the writing of genomic pipelines in popularly used workflow languages and newly emerging community-standards like Nextflow ^12^ and the Common Workflow Language (CWL) ^13^, respectively. While expertise for Nextflow already existed within the network, two collaborators from outside Africa were interested to join the project given their expertise in cloud environments, containerization of code ^14^ and developing CWL ^13^. They subsequently joined the planning and participated in the hackathon. In fact, they were also invited as guest speakers in the network’s monthly webinar series where they shared some of their experiences in these areas with the broader H3ABioNet consortium.

The H3ABioNet Cloud Computing hackathon was announced on the internal H3ABioNet consortium mailing list as a call for interested applicants and in some cases, individuals were invited based on their specific expertise. Most of the participants selected were early career scientists with strong computational skills, an understanding of genomic pipelines and willingness to work in teams. The pipelines for the Cloud Hackathon were divided into four “streams”: 1) Stream A: variant calling from whole genome sequencing (WGS) and whole exome sequencing (WES) data ( https://github.com/h3abionet/h3agatk), 2) Stream B: 16S rDNA Diversity Analysis ( https://github.com/h3abionet/h3abionet16S), 3) Stream C: Genome Wide-association studies (Illumina array data) ( https://github.com/h3abionet/h3agwas) and 4) Stream D: SNP Imputation and phasing using different reference panels ( https://github.com/h3abionet/chipimputation). Successful applicants were given a choice to select a project stream based on their skills and interest- or if unsure, assigned to a specific stream. Streams A and B decided to use CWL for their pipeline development, whereas Streams C and D opted to use Nextflow due to their prior experience using Nextflow.

Stream membership respected participants’ own interests, but it was also sought to have steams of balanced composition. This included bioinformaticians with knowledge in the specific genomic analyses and computational tools required, strong computational skills to create the Docker containers and implement workflows, and strong system administration skills to assist with the installation of numerous software components as needed. We also included bioinformaticians with experience in running the workflows or components of the workflows, and software developers who could assist with creating Docker containers, troubleshoot and implement workflow languages (CWL was still in draft-2 at the time of the hackathon, and some language features were added based on our experience).

To maximize the learning experience, upon selection, participants were given prerequisite tutorials and materials (Github, Nextflow, CWL, Docker and the SOPs) to go through. Communication and planning infrastructure in the form of Slack channels and Trello boards were created beforehand with all the participants added in order to allow them to brainstorm and share ideas with team members before the hackathon began ( Table 2). Fortnightly planning meetings were held starting from 3 months in advance in order for hackathon participants to get involved in planning their proposed tools and to get to know one another and develop a working rapport before the start of the hackathon.

**Table 2.** Communication channels used for the hackathon.

Channel	Link	Purpose
Mailing list	-	Group wide announcements and communications
Mconf	https://mconf.sanren.ac.za/	Online meetings
Slack	https://slack.com/	Inner group discussions and chat
Trello	https://trello.com/	Plan goals and activities, and track progress
GitHub	https://github.com/	Code repository and version control
Google Drive	https://drive.google.com/drive	Document sharing

The hackathon ran in August 2016 and was hosted at the University of Pretoria Bioinformatics and Computational Biology Unit in South Africa. The choice of the hackathon venue was based on the availability of Unix/Linux desktop machines with the facility for sudo/root access enabling participants to install software and deploy Docker containers for testing. Besides the local machines, participants also had access to cloud computing platforms such as Azure and Amazon, Nebula (made available by the National Center for Supercomputing Applications, University of Illinois at Urbana-Champaign), and the African Research Cloud (through a collaboration with the University of Cape Town eResearch initiative). After the hackathon, more testing was also done on EGI Federated Cloud resources (as a courtesy allocation to the University of Khartoum).

### Hackathon week activities

The initial day of the H3ABioNet Cloud Computing hackathon was dedicated to introductions, expectations by the participants and practical tutorials covering the use of CWL, Nextflow and creation of Docker containers to ensure all participants had the same basic level of knowledge. The teams had a breakout session where overall milestones for the streams during the hackathon week were refined, tasks were identified and assigned to team members and Trello boards updated with the specific tasks. Each stream reported back on their progress and overall work plan for the coming hackathon days. For the remaining days of the hackathon, participants were split into their respective streams to work on developing and containerizing their pipelines as well as creating the related documentation. To ensure a successful hackathon with concrete outcomes, the streams spent the first 30 minutes of each hackathon day reviewing their prior progress and updating their Trello boards and reporting to the group what they will be working on. At the end of the day, each stream provided a progress report to the whole group on what they had achieved, what they struggled with and what they will be working on. The start and end of day reporting proved useful as it allowed groups that had encountered and solved an issue to share the implemented solution with another stream, and for different streams to work together to solve any shared issues encountered, thus speeding up the development of the pipelines. Area experts and collaborators would switch between the streams to provide necessary technical expertise.

Communication during the hackathon was facilitated by Slack integration with Trello (for tasks management and progress tracking) and code developed was pushed to GitHub (for live code integration). Table 2 lists the various communication media used during the hackathon. Some groups also utilized Google docs for documenting their progress prior to migrating documentation into GitHub README files.

 Remote participation in the hackathon was facilitated through the MConf conference system. One stream had a participant with very strong coding skills working remotely from the US; who managed to make progress on the corresponding workflow when the other group members were not working due to the big time difference between the USA and South Africa (SA). This ensured continuous development on the workflow when the team in SA would clock off and provide a to-do list which was accomplished by the participant from the US. Noticeable during the hackathon was the team spirit created and the increasingly later end time for the days (with most days ending at 8:30 pm as participants continued working after the different streams provided their daily reports). All participants wished for an extra day or two to complete their pipelines.

### Post-hackathon activities

After the week-long hackathon at the University of Pretoria, members of each stream continued working on their respective pipelines communicating via Slack and Trello. Meetings were held over MConf every two weeks to report on the progress of each pipeline. Upon completion, each group handed their pipeline to other groups to test on different platforms, and thereby avoid bias in implementation and improve the documentation. Consequently, this facilitated the use of the four pipelines developed within H3Africa projects as highlighted in Table 1.

## Discussion

The H3ABioNet Cloud Computing Hackathon was aimed at producing portable, cloud-deployable Docker containers for a variety of bioinformatics workflows including variant calling, 16S rDNA diversity analysis, quality control, genotype calling, and imputation and phasing for genome-wide association studies. The workflows developed in this hackathon benefited from workflow management systems, and further come with Docker recipe files that can be used to build container images when downloading images might be an issue. Thus, Dockerization provided a method to package and manage software, tools and workflows within a portable environment/container, similar to virtualization but with a smaller computing overhead compared to virtualization

The novelty of the H3ABioNet Cloud Computing Hackathon was that all the participants selected were involved in the latter stages of the planning and the setting of some of the outcomes for the hackathon. Critical recommendations during the hackathon planning meetings were that the resulting Docker containers and pipelines developed should be compatible with heterogeneous African research compute environments with portability and good documentation being key. This is especially important considering the fact that access to Cloud computing environments within Africa is still in its infancy. Hence, it was decided that development and testing of the pipelines should occur on a single machine, with the ability to be ported to a cluster or an HPC environment, and ultimately tested and deployed on cloud-based platforms (Amazon, Microsoft Azure, EGI FedCloud, IBM Bluemix, and the new African Research Cloud initiative).

Besides contributing solutions to African problems, three factors contributed to the success of this highly ICT-based activity in an African setting: 1) Almost all the communications tools used ( Table 2) had equivalent apps that work right off a smartphone, a feature that many people within Africa (and less developed countries) tend to make use of ^17^. 2) The used tools were complementary to each other, and integration was sought whenever possible (like between Slack and Trello). 3) The hackathon was timed at the end of the 4th year of the initial H3ABioNet round of funding. At that point, the consortium (via its Infrastructure Working group) had already invested in improving the computational infrastructure within the network ^11^, including tools for regular communications and webinars ^18^. In a sense, Table 2 also represents our vetted list of collaborative tools in the light of 4 years of feedback from the consortium.

## Lessons learnt and concluding remarks

The opportunity to link people physically and focus solely on one project has been highly effective in providing the main outline and proof of concept outputs. However, once people were back home, continuing the tasks has been a challenge. Clearly defining the roles and commitment of all the participants in the papers reporting the results should encourage them to complete the work, and increase their accountability.

The communication and management tools used for this hackathon ( Table 2) were important as these tools facilitated interaction between and across team members and enabled the participants to continue to work in a structured manner once back at their respective institutions, despite time zones differences.

The H3ABioNet Cloud Computing Hackathon has been an important milestone for the Network as it brought together people with various skills to work on focused projects. It signalled the shift from capacity building to utilizing the capacity developed in order to tackle problems specific to the heterogeneous African computing environments, as defined and implemented by the mostly African participants. Equally important, this hackathon was not done in isolation from the rest of the scientific community nor could it have succeeded without local collaborations. This aspect, i.e. welcoming input and actively seeking it when needed from outside the consortium, is key to truly empowering the local community.

As software packages and computing environments evolve with varying build cycles and new bioinformatics tools become available, we envision that hackathons to keep these pipelines current, adopt new technology implementations such as Singularity, and develop new workflows such as for RNA-Seq analysis will occur. The pipelines developed during the H3ABioNet Cloud Computing hackathon will be used for training and data analyses for intermediate level bioinformatics workshops, and for scientific collaborations requiring bioinformatics expertise for data analysis such as with the H3Africa genotyping chip and GWAS analyses. Future H3ABioNet hackathons would also provide an opportunity to utilize the skills of trained bioinformaticians at intermediate and advanced levels, who would not otherwise attend bioinformatics training workshops, to come together to derive practical solutions that are of benefit to the African and wider scientific community.

## Data and software availability

All data underlying the results are available as part of the article and no additional source data are required.

The four pipelines are available publicly via H3ABioNet’s GitHub organization page https://github.com/h3abionet in the following repositories: (h3agwas, chipimputation, h3agatk and h3abionet16S) as well as container images on quay.io at quay.io/organization/h3abionet_org and dockstore at: https://dockstore.org/workflows/h3abionet/h3agatk

All code is available under MIT license.
