# Supplementary material for: Organizing and running bioinformatics hackathons within Africa: The H3ABioNet cloud computing experience
Source: AAS Open Res. 2019 Aug 7;1:9. Originally published 2018 Apr 18. [Version 2] doi: 10.12688/aasopenres.12847.2 (PMC7194140; doi:10.12688/aasopenres.12847.2)
Supplement: Peer review history

# Peer review: Reviewer response for version 2

**DOI:** 10.21956/aasopenres.14070.r27114

**Published:** 2019-09-19

**Authors:** Juan Ruiz-Alzola

**Affiliations:** 1 Institute for Biomedical and Health Research, University of Las Palmas de Gran Canaria, Las Palmas de Gran Canaria, Spain

The authors have improved their original submission. In my view the article is now ready to be approved.

I confirm that I have read this submission and believe that I have an appropriate level of expertise to confirm that it is of an acceptable scientific standard.

# Peer review: Reviewer response for version 2

**DOI:** 10.21956/aasopenres.14070.r27113

**Published:** 2019-09-04

**Authors:** Steffen Möller

**Affiliations:** 1 Institute for Biostatistics and Informatics in Medicine and Ageing Research, Rostock University Medical Center, Rostock, Germany

Well done!

I confirm that I have read this submission and believe that I have an appropriate level of expertise to confirm that it is of an acceptable scientific standard.

# Peer review: Reviewer response for version 1

**DOI:** 10.21956/aasopenres.13913.r26312

**Published:** 2018-06-22

**Authors:** C. Titus Brown, Rayna Harris

**Affiliations:** 1 Department of Population Health and Reproduction, University of California, Davis, Davis, CA, USA

**Summary and impression**

 The article describes the organization and execution of a hackathon in Africa with the goal of producing cloud-deployable Docker containers for four bioinformatic workflows. 

 The article begins with a brief history of H3ABioNet and the need for bioinformatics infrastructure, training, and community in Africa. 

 Then the authors describe their pre-hackathon preparations, the weeks of activities during the hackathon, and their lessons learnt. No quantitative assessment of the hackathon was provided, but the authors do provide links to the bioinformatic workflows used. 

 Hackathons are an increasingly popular way of building tools and community for research and education. This article nicely describes why a hackathon was needed in Africa and how the authors went about organizing and executing their first hackathon. The article contains some useful suggestions for what bioinformatic or social tools can be used to facilitate research and communication. I think it is important to communicate this type of activity to the broader scientific community, however, I have a few concerns. 

**Major issue**

 My main concern is that this paper was submitted as a "method article". My understanding is that methods articles should describe new empirical or computational methods that are described in sufficient detail such that they can be reproduced. While this article does describe the authors' strategy for organizing and running a hackathon, this article is written more as a retrospective piece on the event rather than a recipe for running a hackathon. I am not convinced that it should be published as a method paper; unfortunately, there doesn't appear to be a more suitable platform within the AAS journal.  

 https://aasopenresearch.org/for-authors/article-guidelines/method-articles 

**Minor issues**

1. The figure legend does include a title, but it does not include a description of the key points nor does it explain the meaning of the arrows. According to the AAS guidelines, "the legend should be sufficiently detailed so that it can stand alone from the main text". Additionally, it is not clear from first glance that "4 portable computational workflows" is the goal of the hackathon. This could be made more clear.
2. Table 1 provides a nice overview of communication channels. Can you elaborate and add what tools you used for sharing documents (e.g notes, slides, pdf)?
3. On page 4, the authors state: "Vital in setting up the teams...". Does this paragraph refer to the expertise of the learners/participants or to the people who are leading the stream or both?
4. On page 5, the first sentence of the discussion provides the first clear statement of the aim of the hackathon (in my opinion). The goal is mentioned in the abstract and intro, but it isn't as clear. It is in this paragraph that I realized that you had working pipelines, but they were not "dockerized". After reading this, the figure made a lot more sense. I recommend revising the abstract and intro to make it clear what the starting point (5 bioinformatic workflows not in the cloud) and the endpoints (4 bioinformatic workflows in the cloud).
5. The paragraph on "Post-hackathon feedback and actions" seems incomplete or perhaps is mislabelled. This paragraph describes communication and work that extended past the hackathon, but it does not describe any assessment or feedback mechanisms that were used. Also, what happened after groups traded platforms? Was the documentation improved or was this simply the goal?
6. The article jumps from "Introduction" to "Discussion" without providing a clear a description of some of the results or outcomes. Is there a reason why some statistics regarding participation or progress toward the goal are not reported? If there is a reason why demographic information cannot be provided, that is fine, but a report about how much progress was made toward the goal of dockerization would be useful.
7. While I appreciate that you provided a link to the GitHub repo and Docker container, I highly recommend getting a DOI for these repositories. The AAS data guidelines page provides a list of providers. Some of your GitHub repositories already have versions, so it should be fairly easy to import these into Zenodo for a DOI. https://aasopenresearch.org/for-authors/data-guidelines.

We confirm that we have read this submission and believe that we have an appropriate level of expertise to confirm that it is of an acceptable scientific standard, however we have significant reservations, as outlined above.

# Peer review: Reviewer response for version 1

**DOI:** 10.21956/aasopenres.13913.r26376

**Published:** 2018-05-02

**Authors:** Juan Ruiz-Alzola

**Affiliations:** 1 Institute for Biomedical and Health Research, University of Las Palmas de Gran Canaria, Las Palmas de Gran Canaria, Spain

I have overall enjoyed reading this paper. The implementation of the hackathon is well explained, and provides good hints for others to replicate both in Africa and in the rest of the World. In particular, a large array of ICT-based collaborative tools has been applied whose use is very interesting everywhere. The motivation is also very relevant, as hands-on knowledge sharing is important to build distributed strong scientific communities that can develop new solutions and keep collaboration active within wide multi-site networks. This is particularly so in the case of bioinformatics, a profoundly interdisciplinary and complex field, key to modern knowledge-based economy, health and welfare, where Africa could leverage important opportunities as far as some hurdles are properly managed. It is in this framework where I see the main contribution of this paper, and I must point out the relevance of the collaboration among African and rest of the World institutions and researchers, in addition to the internal African collaboration. In my view all of this is essential to unlock all the potential, and achieve not only scientific success but a wider interaction of great value for economic development. I’m not getting into any technical detail of the pipelines since, as reported in the paper, they will be presented elsewhere.

 Nevertheless I have some constructive criticisms that I’d like to share:

1. I’ve pointed out that the rationale is only partly explained essentially because some boundary conditions should be explained: how many nodes and researchers are participating, what their access to Internet is like in their sites, what difficulties they’re experiencing in their home institutions to access to knowledge and to interact with other international colleagues, etc. Africa is large with very different situations across countries and within each country. It’d be good that the reader could understand better the everyday situation that motivates an action like this, or a wider program such as H3ABioNet. It’d also be good to explain a bit longer what H3A and H3ABioNet are, and how they become important programs for the scientific development of Africa.
2. The description of the method is technically sound, but I have some concerns about how realistic it is to expect such sophisticated ICT-based collaboration, difficult anywhere, considering the existing difficulties for high bandwidth connections in many areas of Africa, including many Universities in main cities. Our group is currently involved in a cooperation with research groups in four African countries. Internet access, good enough for sharing medical datasets, for example, or just to hold videoconferences, is something that we cannot take for granted. Again, this is also related to (1) and boundary conditions, but it is my experience that dealing with this sort of issues is key for success and, at least, to replicate the experience elsewhere.
3. The conclusions are somehow conditioned by what I’ve already pointed out.  

 

 In summary, I think this is a very interesting and motivating paper that could benefit of a better explanation of the boundary conditions, and of how the specific difficulties have been overcome, so that others can replicate the experience in the same or in different fields across the continent.

I confirm that I have read this submission and believe that I have an appropriate level of expertise to confirm that it is of an acceptable scientific standard, however I have significant reservations, as outlined above.

# Peer review: Reviewer response for version 1

**DOI:** 10.21956/aasopenres.13913.r26315

**Published:** 2018-04-18

**Authors:** Steffen Möller

**Affiliations:** 1 Institute for Biostatistics and Informatics in Medicine and Ageing Research, Rostock University Medical Center, Rostock, Germany

The paper describes an in-person meeting in Africa to further develop workflows for genomic analyses and distribute that skill throughout the continent. As a Northern European I can only remotely assess the difficulties of computational biology and bioinformatics services in Africa in local areas. I can see how important such Hackathons are to edutRain research groups with difficulties to attend international conferences. And, with some experience in attending and organizing such events, I was very curious about what may be different in Africa. After all, in Europe there are communities having difficulties to commute, too. The European Union has extra funds for graduate students from Eastern European countries for instance. But there are also highly talented high school students who are under age or do not have the funds or scientific contacts to travel/be trained. So, if you have developed principles for African talents to overcome such obstacles to learn about such Hackathons and prepare for them, then I would be very much interested to see what we can adopt over here.

 From what I then read in the article, there was not so much that the authors did differently, except that they seem to have done it particularly well. Table 1 describes a whole bunch of communication channels when typically it is a mere Wiki or co-editing site orchestrating the participants. Still, every attendee had to physically attend, except that the event was in Africa describing an African set of resources and there was external expertise flown in. I tend to think that here the event fell short of what could have been (and is often) done to invite remote participation. Also, one could videograph training material to support the further distribution of these Africa-specific analysis skills.

 I am also a bit critical about the dominance of online resources that demand good internet connections as in the download of gigabytes of data: Docker. Here I wish more would be done to promote offline services. But I am biased, the authors NM and MRC know me as a contributor to Debian Med, MRC being a Debian contributor himself, which basically means that participants could take a DVD home and perform analyses of their samples without Internet access involving as many local-to-them machines as they like. For the workflows one doesn’t need most of the complicated bits for which one needs computational expertise the article is describing. And that may have helped the post-Hackathon drop in participation, while, of course every event sees that drop and that is a main reason to have such a dedicated time to jointly develop our research environments in the first place.

 Concerning the scientific results, I understand that there is a separate paper prepared. Still, can you say as much as if there are scientific papers out there that already employed the pipelines for their analysis? I mean, from the time before the Hackathon? This would emphasize that you are indeed redistributing a very current set of skills with practical acceptance in the community.

 There is something else to it all. Hackathons form a social network of trust. And you need trust in locally well-described samples. The genetic diversity of Africa is a gem, but one needs to be aware of the demands for population stratification. And because of the harsh environment conditions, one can expect considerable batch effects on samples taken, for which the emphasis on equally collected control samples is important. You can certainly read about it all, but it will help to hear in person about that study that was ruined because cases and controls were kept separate and one box had the dry ice evaporated early – factor variation and confounding technical parameters cannot be communicated enough. Well-established workflows allow participants to analyse their local data independently, have extra parameters matching local concerns, with matching local controls, which will all improve the quality of the pan-African study at large.

 So, I do not think that anything performed for the organisation of this Hackathon was specific to Africa. In the contrary, there should have been some teleconferencing to it. The “initial day to get everyone up to speed” reminds me a bit more of a Summer School than a Hackathon, for which it is not uncommon that most participants already know each other for some time and would not need that day. Could you possible elude a bit more on how the work performed was structured? And how was the external expertise intermingled with your local needs for Africa?

 I would want to retitle the paper towards something like “Hackathon on workflows for genomics held in Africa”. The H3ABioNet workflows are nicely accepted everywhere, I tend to think, so, why not have an H3ABioNet workflow Hackathon in Europe with some Africans flying in? Clarifying that a bit more may help the paper.

I confirm that I have read this submission and believe that I have an appropriate level of expertise to confirm that it is of an acceptable scientific standard, however I have significant reservations, as outlined above.

# Response

**Published:** 2018-04-21

**Authors:** Victor Jongeneel

**Affiliations:** Institute for Genomic Biology, University of Illinois at Urbana-Champaign, USA

Thanks very much to Steffen Möller for his pointed comments. It is certainly true that this hackathon did not differ in any major way from similar events held in high-income countries, and did not incorporate any features specific to the African context. Its primary value was to expose African scientists to the practical aspects of community development of computer code, and to try to create a community around the maintenance of a set of workflows that implement methods that are useful to the H3Africa research community and beyond.

There are aspects of the work that may not have come across in the paper. For one thing, the workflows implementing haplotyping, imputation, and GWAS analysis were based on work done in the framework of the H3Africa AWIGen project, and are in production for the analysis of data generated by this project. Similarly, the workflow for variant calling from WGS data was used in the analysis of 350 African genomes that has led to the design of a novel genotyping chip optimized for African populations, and the 16S rDNA sequence analysis was derived from work done to analyze bacterial populations present in leg ulcers of sickle cell patients in Nigeria. Therefore, all of the code developed during the hackathon was solidly anchored in existing genomic analysis projects in Africa.

Secondly, the workflows developed in the hackathon serve as practical implementations of Standard Operating Procedures for the H3Africa Accreditation Exercises, which are used to evaluate the capacity of African research groups to analyze complex genomic datasets being produced by its research projects (see Jongeneel et al, PLoS Comput Biol, PMC5453403). Success in taking one of the exercises is considered a landmark for African groups who are preparing to step into the existing gap between data production and data analysis, where the analysis is typically undertaken by First World groups.

It is true that the authors, including myself, could have done a better job at explaining how the hackathon and its products are anchored in the H3Africa research ecosystem. I hope that the above clarifies this.

As a final remark, while highly Internet-dependent tools were used extensively during the hackathon, to my knowledge none required a very high bandwidth. At least two of the participants attended remotely from North America, and were able to contribute substantially in part because of their time zone differences and asynchronous contributions.
